# Perpetuating global inequalities in the knowledge economy: The case of HIV social science research in East Africa

**DOI:** 10.1080/17441692.2025.2466731

**Published:** 2025-02-20

**Authors:** Daniel Wight

**Affiliations:** MRC/CSO Social and Public Health Sciences Unit, University of Glasgow, Glasgow, UK

**Keywords:** Global health, research capacity strengthening, HIV, Africa, dependency theory, SDG 3: Good health and well-being, SDG 4: Quality education, SDG 10: Reduced inequalities, SDG 16: Peace, justice and strong institutions

## Abstract

Despite almost a century’s research capacity strengthening in Africa, HIV/AIDS research has been dominated by high-income countries (HICs), illustrating broader inequalities in the global knowledge economy. The perpetuation of weak social science capacity in east Africa is analysed as part of a complex system with multiple causes at different socio-ecological levels. Furthermore, although primarily driven by HIC/ neo-colonialist interests, causes also stem from low-income countries (LICs), and individual actions reproduce macro-level structures. Most factors link to global economic inequalities, and the extraction of data and intellectual capacity from east Africa operates akin to Dependency Theory, but this is exacerbated by African governments. At the meso-level, HIC institutions prioritise revenue and publications over strengthening LIC research capacity, whatever their rhetoric, while serious impediments exist in east African institutions. At the micro-level, HIC researchers perpetuate inequalities through, e.g., prioritising output, maintaining dependency, and choosing HIC rather than LIC conferences and journals. Multiple responses are needed, particularly at the macro-level, especially long-term, tailored funding. Meso-level responses include meritocratic career structures and institutional research consultancies. Individual HIC researchers should, ideally, prioritise training and mentoring, but this risks career advancement. Above all, honesty is required about motives and conflicting interests, at institutional and individual levels.

## Background

In 1988 I, a Briton, was interviewed to lead a social science programme in a new Medical Research Council (MRC) research unit on HIV/AIDS in Uganda. Happily, someone far better qualified was appointed, but I was perplexed as to why I was being interviewed at all. I had never worked on HIV/AIDS or been to east Africa, knew nothing about the culture and spoke no Luganda, the dominant local language. Since then, there have been at least four rounds of recruitment to lead the Unit’s social science programme, in each case resulting in a British, Dutch or American appointment. This is particularly striking given the MRC Unit’s frequently stated goal of strengthening Ugandan research capacity and Uganda’s renowned Makerere University generating around a thousand social science graduates each year.

When I did come to work on HIV in east Africa in the 1990s, I learnt that this specific example was indicative of a much wider phenomenon. Despite eastern/central Africa being at the core of the HIV/AIDS pandemic, nearly all large-scale research programmes on HIV/AIDS were led from high income countries (HICs), less than 15% of scientific publications on HIV came from Africa and only a third of those were led by African authors (Yach & Kenya, 1992). Furthermore, major debates on African sexuality were led by HIC academics (e.g. Caldwell et al., 1989). This illustrates a much broader problem which largely persists today. In most African countries scientific institutions remain fragile, severely under-resourced, and generated only around 1% of the world’s science publications in 2000 (Rahman & Fukui, 2003) and about 3% in 2016 (Beaudry et al., 2018).

There are many interrelated consequences of this globally distorted pattern of research, ranging from practical challenges, such as ex-patriate researchers’ limited understanding of local culture or language, through implications for public engagement, such as local mistrust of studies and their findings, to fundamental epistemic and political implications. In particular, it restricts intellectual and therapeutic sovereignty, with HICs defining the problems of, and solutions for, LICs (Chu et al., 2014; Cleland & Watkins, 2006; Wintrup, 2022). Similar knowledge-power dynamics, generating ‘epistemic injustice’, have been analysed within health inequalities research on race in the U.S.A. (Petteway, 2023). Broadening beyond health research, there are now several critiques of a Euro-American hegemony over the social sciences, variously promoting epistemologies (Santos & Meneses, 2009) or theories (Comaroff & Comaroff, 2011; Connell, 2007) of ‘the South’. This literature is beyond the scope of this commentary, but it has been critically reviewed by Rosa (2014).

The global inequalities in health research can have profound consequences, such as slow or ineffective responses to public health crises in Africa (Cleland & Watkins, 2006), fragmenting health systems (Wintrup, 2022) and distrust of foreign experts (Leach, 2010). Although South Africa’s research output is now exceptionally strong for sub-Saharan Africa (Collyer et al., 2019; Mouton et al., 2019) a tragic example of distrust comes from that country in 2001. President Mbeki claimed that HIV agencies are ‘Convinced that we are but natural-born, promiscuous carriers of germs,.. [with] unconquerable devotion to the sin of lust' (The Mail and Guardian, 2001) and rejected their recommendations for HIV treatment, with devastating consequences for generations of South Africans.

Why is social health research in east Africa still dominated by HICs despite: recognition since the 1920s that indigenous research capacity is essential for development (Rockefeller Foundation, 1962); over half a century of funding from HICs to east Africa to develop this capacity; numerous practical WHO initiatives; and recurrent analysis of the problem in medical journals since the 1990s? The question is important for global health politically, ethically and epistemologically. A recent critical review on ‘decolonising’ global health argues that ‘ensuring proper representation of, respect for, and accountability to, “Southern” policymakers, researchers and practitioners … will require a properly formulated diagnoses of the problem … ’ (Hellowell & Nayna Schwerdtle, 2022). This paper outlines a model of how inequalities are perpetuated in the global knowledge economy from the perspective of involvement in HIV research in east Africa since the 1990s. I argue that, like most intractable problems, this is part of a complex system with multiple causes located at different, inter-related socio-ecological levels. The extent to which it is a deterministic model depends on the relative importance of the socio-ecological levels: while micro-level individual actions often reproduce and reinforce macro-level structures, they can also modify them (Giddens, 1984). Furthermore, the causal factors are not all driven by the interests of HICs/ neo-colonialists (Khan et al., 2021). However, as with all models, this depiction is partial and cannot fully represent the complexities and nuances of real life.

## Macro-level

### HIC drivers

**Figure. 1.**
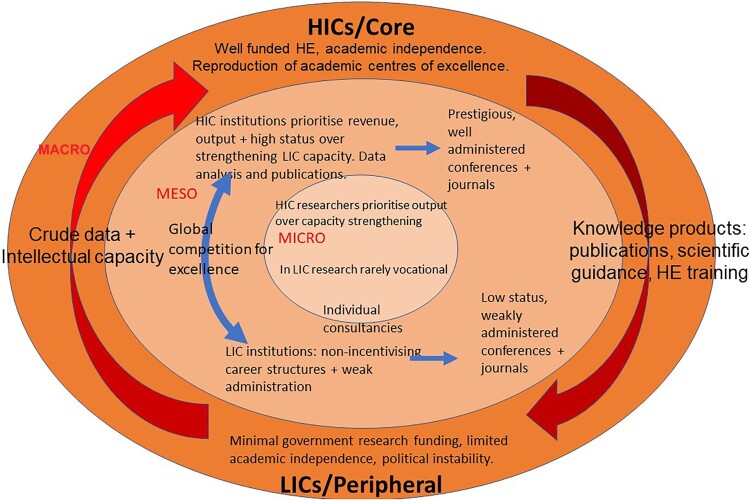
Macro, meso and micro factors perpetuating inequalities in the global knowledge economy.

At the macro level, global economic inequality is fundamental (Ojanperä et al., 2017). In LICs poor quality education and teacher training, courses chosen for vocational reasons, university mismanagement and corruption, minimal research funding, and the drain of expertise to HICs are largely attributable to economic adversity (Nchinda, 2002; Pang et al., 2002; Sall, 2003; Sitthi-amorn & Somrongthong, 2000; Zeleza, 2003). Dependency Theory’s analysis of differential industrialisation to explain global economic inequality (e.g. Boshoff, 2009; Frank, 1967; Kvangraven, 2020; Prebisch, 1950; Watson, 2012) can be applied to the global knowledge economy. Thus the extraction of raw materials from ‘peripheral’ LICs to ‘centre/core’ HICs is akin to research data (Chu et al., 2014; Khan et al., 2021), the added value through manufacturing is akin to analysis and academic publication, the accumulation of manufacturing capacity is akin to the brain drain (Lazarus et al., 2010; Mouton & Waast, 2009), and the sale of manufactured products back to the periphery is akin to the provision of publications, scientific guidance and higher education training from HICs to LICs (Figure 1).

Important to the UK’s powerful role in global health research is the government’s use of overseas aid to support British universities, with research funding overwhelmingly awarded to UK institutions since the 1990s. In 2015 the new Global Challenge Research Fund’s research agendas were driven by UK, not LIC, researchers, blue sky thinking was preferred to applied research, lack of LIC experience was welcomed, and initially there was no scope to strengthen LIC research capacity. ‘Challenges’ identified in a rapid review included: inadequate impact; unstructured research capacity building; MICs being the main beneficiaries; and, possibly, this being tied aid (Independent Commission for Aid Impact, 2017).

### LIC drivers

However, to believe in a model of the global knowledge economy that posits that the stark inequalities are entirely driven by HICs exploiting and reinforcing their advantages over LICs would be naïve (Khan et al., 2021). Sub-Saharan countries only contribute 0.4% of GDP to scientific research compared to 2.4% in Europe (World Bank, 2017), and of the ten main funders of science in Africa only two are African (Beaudry et al., 2018). Furthermore, there is a growing literature on the role of African governments and elites in ‘underdeveloping Africa’ and impoverishing the continent (Emeh, 2013; Igwe, 2010; Ope-Agbe, 2007). In many African countries unstable governments, poor civic structures and lack of academic independence have led African social scientists to flee to America or Europe (Beaudry et al., 2018). Universities are often seen as a political threat and there is little investment in higher education and research (Gaillard, 2003).

## Meso-level

### HIC drivers

Universities are central to the global knowledge economy (Altbach, 2007; Olssen & Peters, 2005) with ‘centres of excellence’ overwhelmingly in HICs (Altbach, 2007) and African universities characterised by poor research infrastructure and management (Ezeh et al., 2010; The Academy of Medical Sciences, 2012). HIC universities frequently present their ‘internationalisation’ initiatives, whether enrolling LIC students or conducting overseas research, as contributing to international development and strengthening LIC research capacity (e.g. University of Glasgow, 2007). However, given the increasingly marketised and competitive nature of higher education in HICs, it is understandable that universities’ primary concerns are to boost revenue and prestige.

Overseas student fees comprise approximately 19% of total UK university income (Universities UK, 2022) generating £26 billion annual net benefit to the UK economy (London Economics, 2021). To maximise this income it is in universities’ interests to relax English language entry standards, exaggerate the relevance of courses for LMIC contexts, increase the postgraduate student: supervisor ratio, and relax exam grading. Governance-preventative mechanisms are not always adequate. Though most overseas students return, studying in HICs, particularly at post-graduate level, fuels the brain drain by providing HIC contacts, experience and expectations.

Many funders try to make support for HIC-LIC research collaborations conditional on the active involvement of LIC partners, but given structural inequalities this frequently fails. HIC funding bodies often prioritise theoretically ‘cutting-edge’ research over addressing problems identified within the LIC, and requirements that proposals are led by LIC institutions can be subverted by HIC institutions that drive the collaboration (e.g. Wellcome Trust, 2009). Other imbalances in HIC-LIC collaborations can include: poaching the best local researchers at the cost of locally-funded studies (Chu et al., 2014); the isolation of these researchers from their LIC colleagues; study-specific development of LIC research capacity, rather than meeting long-term needs; prioritising academic publications over implementation of findings; and authorship guidelines (see Micro-level below) (Jentsch & Pilley, 2003).

Although the literature on research capacity building emphasises the importance of institutional capacity (Berg, 1993; Potter & Brough, 2004), several factors counter such strengthening. In a competitive global knowledge economy it is not in the interests of HIC universities to substantially strengthen the capacity of LIC competitors. In HIC-LIC collaborations differing administrative and management practices and standards can be challenging, sometimes prompting HIC institutions to establish new research institutions in LICs that parallel existing structures (e.g. the Mwanza Interventions Trials Unit, Tanzania, alongside the National Institute of Medical Research). These sidestep administrative difficulties but can undermine existing LIC institutions, for instance by poaching their best staff. (Ex-)colonial governments and NGOs have funded research centres in Africa since before the Second World War (Rockefeller Foundation, 1962) which are largely independent of local research institutions. They have generated much important research, trained and employed many local scientists and contributed to the local research culture. However, they have long been criticised as ‘semicolonial’ ‘annexed sites’ (Costello & Zumla, 2000), and in the last decade, several have strengthened formal ties with British, rather than local, universities (e.g. African MRC Units with LSHTM).

Prior to COVID19, two major HIV/AIDS international conferences encapsulated the gulf between the research worlds of HICs and Africa, and the processes perpetuating this. The International AIDS Society (IAS) biennial conferences became the ‘largest conference on any global health issue in the world’ (WHO, 2008) with over 15,000 delegates. They were very well organised, attracted the world’s leading HIV researchers, commanded enormous press interest, and had an important advocacy function, making them influential networking hubs in the international HIV research industry. They were also extremely expensive (US$ 600–1000 in 2018 + food and accommodation). While many LIC delegates lacked funds to attend, the most expensive hotels were often booked-up first. The conferences boosted their host cities’ economies yet 18/22 were held in HICs, none in LICs, and it was 2000 before any were held in Africa.

By contrast, the International Conference on AIDS and STIs in Africa was always in Africa, organisation was often chaotic, reflecting little investment in administrative capacity in Africa, presentations were of variable quality, and it was relatively cheap. Leading scientists rarely attended, especially those from HICs. This was self-perpetuating, given conferences’ networking role: influencing key people, exercising and maintaining power. Research institutions and individual researchers’ decisions about which conferences to attend reinforce global inequalities, whatever the IAS rhetoric to modify them.

### LIC drivers

However, problems within African research institutions also perpetuate limited capacity and are a main driver for HIC partners to create parallel structures. Lack of career paths for good researchers has been identified as ‘the most serious impediment to health research’ in Africa (Whitworth et al., 2008), and in many institutions, the careers of tenured staff are only weakly linked to research output and expertise. Patronage and ethnic loyalties can undermine meritocratic promotion, while very low salaries encourage the perspective widespread in state organisations that: ‘You pretend to pay us; we pretend to work’. A combination of poor training, limited career incentives, and Euro-American researchers' obligation to collaborate with LIC institutions can result in LIC research institutes contributing little intellectually to research agendas, design or analysis. These challenges can contribute to unsupportive cultures, with little mentoring, limited intellectual debate, junior staff conducting much of the work, and systems of deference and patronage. Further, research governance can be weak and/or excessively cumbersome, with little scrutiny of publications (Chu et al., 2014).

An important contributor to limited research capacity is that much social science research in Africa takes the form of small, individually contracted consultancy projects for NGOs and government departments (Gaillard, 2003; Wight et al., 2014). This is driven by institution’s low monthly salaries (Gaillard, 2003), sometimes close to a day’s consultancy fee. A Uganda study found that consultancies are seen to constrain professional development and institutional research capacity, prevent the development of research specialisms and compete with teaching and supervision (Wight et al., 2014). Meanwhile, research commissioners are broadly satisfied with the research expertise available, feel no responsibility to strengthen capacity, and the potential of institutional consultancies is under-developed.

## Micro-level

At the micro-level global health researchers are implicated in the inequitable knowledge system that many are trying to revise. In particular, our material interests, largely shaped by differing career structures in high- and low-income countries, can lead us to reproduce inequitable structures through our individual actions.

### HIC drivers

Living in the country enables a better understanding of context than flying in for ‘helicopter’ research. However, practical constraints in LICs mean Euro-American researchers, even with egalitarian ideals, often default to lifestyles that, in important respects, parallel those of junior colonial officers half a century earlier. Similarities include housing, European diet, domestic staff, elite transport, private healthcare, contact with locals limited to domestic staff and colleagues, and overwhelmingly ex-patriate social life and cultural references. This distances them from the experiences and perspectives of their LIC colleagues, let alone those they are studying and/or intervening with, and undermines balanced collaboration (Kulesa & Brantuo, 2021). Although working in LICs involves hardships, income tax exemption, allowances and cheap domestic staff can make it financially attractive.

HIC researchers’ dependence on research funding, successful projects and publications for career advancement, rather than mentorship and capacity strengthening, can undermine long-term LIC capacity strengthening in several ways (Chu et al., 2014). Output is optimised through meritocratic appointments, frequently advantaging Euro-Americans, with African researchers often hired primarily for data collection and transcription, rather than as intellectual partners. This can marginalise African researchers’ understanding of the research context and their interpretation of the data. Conversely, funders’ requirements for HIC-LIC collaborations commonly lead to partnerships with LIC researchers with inappropriate expertise and minimal input to proposals. Subsequently, the need to meet deadlines and maximise publications encourages HIC partners to fulfil research tasks themselves, rather than support their LIC colleagues to do so, since this is generally far quicker but perpetuates dependency. The Medical Journal Editors’ authorship criteria (a meso-level factor) privilege intellectual input over fieldwork or local knowledge, benefiting HIC researchers. Although often contentious within collaborations (Chu et al., 2014; Jentsch & Pilley, 2003), new guidelines are rarely negotiated, in part because very time-consuming, Miles et al. (2021) being a notable exception. To meet LIC researchers’ expectations and encourage future collaborations, HIC lead researchers sometimes grant them token authorships (Chaccour, 2018; Chu et al., 2014). This has the unintended consequence of undermining the credentials of LIC researchers who do make a substantial intellectual contribution.

### LIC drivers

For African researchers, exceptional motivation and sacrifice are often necessary to develop internationally recognised expertise, given economic insecurity, limited career paths and opportunities for global exposure, and institutional patronage. For many, if not most, research is not vocational; the imperative is to maximise income, whether through consultancies or changing occupation altogether (Gaillard, 2003). This encourages highly individualistic and short-term perspectives, with little loyalty to research teams, projects or departments. With much research conducted through individual consultancies, ‘*Most of our social scientists are not institution based … They are there for hire*’ *(Faculty dean)* (Gaillard, 2003; Wight, 2008). There is little reward for extra intellectual effort and appeasing superiors can be a higher priority than publishing. Many researchers attribute their lack of publications to not being funded to write: they generally have less scope for this outside contractual hours than do HIC academics. Given these constraints, it is understandable that few take the risk of challenging the Euro-American-centric social science establishment (Rosa, 2014).

Funding bodies’ requirement that HIC researchers collaborate with in-country institutions for LIC research gives established LIC researchers a strong bargaining position. They receive frequent invitations to collaborate, bringing institutional and/or personal funding, and it is in their HIC partners’ interest to disguise any imbalance in intellectual or managerial input. Consequently, senior LIC academics are sometimes investigators on a dozen or so large international grants, making modest contributions and rarely taking the opportunity to shape the research agenda, but augmenting their salaries. These collaborations can compete with teaching or supervision.

## Conclusions and recommendations

Like most persistent problems, weak research capacity in east Africa can be viewed as a feature of a complex system. I have tried to identify key factors in the global knowledge economy that perpetuate inequalities despite numerous attempts to strengthen capacity over half a century. They exist at every level of the system and, critically, are not only driven by HICs, institutions and researchers (Khan et al., 2021). I was alerted to many of the east African drivers noted here by east African colleagues. Most factors link back to global economic inequalities and the extraction of data and intellectual capacity from LICs, analogous to economic Dependency Theory, but they are sometimes exacerbated by African governments. Numerous factors operate at the meso-level, with HIC institutions prioritising revenue, research funding and publications over strengthening LIC research capacity, whatever their rhetoric. Meanwhile, in east Africa there are serious institutional impediments to strengthening capacity, especially the lack of career structures that incentivise research excellence. At the micro-level, HIC global health researchers have greater agency than east African researchers, perpetuating inequalities through, for instance: prioritising our own output over mentoring LIC colleagues; shouldering research management and maintaining dependency (Chaccour, 2018); prioritising immediate project success over long-term capacity strengthening; and choosing HIC rather than LIC conferences and journals.

However, this argument has not acknowledged many commonalities between HICs and east Africa, especially in higher education. Nor has it documented many important initiatives to address the problem, such as the African Population and Health Research Centre, Nairobi, and some exceptional models of balanced, mutually beneficial collaboration. It would be more nuanced, and probably more realistic, to present east African and HIC circumstances as at different ends of a continuum, rather than binary opposites, and to recognise power imbalances within HICs and east Africa. Furthermore, although they have more limited agency than HIC researchers, east African researchers can, to some extent, modify macro-level structural factors. As Collyer et al. (2019) argue: ‘ … the flow of knowledge and resources can be negotiated, thus producing spaces for contestation and forms of ‘local knowledge’ (p. 86). While some HIC research institutions have reinforced their dominance in Africa in the last decade, there are also examples of increasing research capacity in east Africa, demonstrating that the interplay between different macro, meso and micro factors is not static.

Intractable problems usually require multiple responses at different levels, higher level interventions generally being most effective. A useful start would be clearly defined indicators for capacity strengthening at national, institutional and individual levels (Nurse & Wight, 2011). At the macro-level long-term, predictable funding tailored to specific local environments is needed (Chataway et al., 2005), and east African countries should have far greater control in shaping capacity strengthening initiatives (Chu et al., 2014), although fundamental goals might not be shared.

At the meso-level, the benefits of strengthening institutional rather than individual capacity have long been recognised (Berg, 1993: Potter & Brough, 2004). Creating attractive, meritocratic career structures that incentivise intellectual effort is a pre-requisite (Whitworth et al., 2008). Establishing institutional rather than individual research consultancies could improve output and accountability, with institutional overheads paying for: dissemination of reports, improved infrastructure, writing time and training (Wight et al., 2014). Hybrid post-graduate degrees between east African and HIC universities would harness the attraction of high status HIC universities while retaining the most talented east African students in east African universities. In the long-term hybrid degrees might contribute to higher standards of supervision in east African universities and redistribute research excellence. Strengthening institutional capacity would, ideally, improve administrative capacity to organise international conferences. More directly, well-resourced, prestigious academic societies in HICs might partner with equivalent organisations in LICs to strengthen their capacity to organise efficiently-run, high calibre conferences.

At the individual level HIC researchers should, ideally, devote more time to training and mentoring, not condone token authorship, prioritise LIC conferences and journals, and support LIC colleagues to secure their own research funding. However, such activities would often be at the cost of career advancement. We need to be honest about our motives and conflicting interests, both those for ourselves and our institutions (Chaccour, 2018; Khan et al., 2021).
